# HDL cholesterol efflux normalised to apoA-I is associated with future development of type 2 diabetes: from the CORDIOPREV trial

**DOI:** 10.1038/s41598-017-12678-9

**Published:** 2017-10-02

**Authors:** Ruth Blanco-Rojo, Pablo Perez-Martinez, Javier Lopez-Moreno, Javier Martinez-Botas, Javier Delgado-Lista, Ben van-Ommen, Elena Yubero-Serrano, Antonio Camargo, Jose M. Ordovas, Francisco Perez-Jimenez, Diego Gomez-Coronado, Jose Lopez-Miranda

**Affiliations:** 1 Lipids and Atherosclerosis Unit, UGC Internal Medicine, Reina Sofia University Hospital, Cordoba, Spain; 2 Nutrigenomics and Metabolic Syndrome Group, Maimonides Institute for Biomedical Research at Cordoba (IMIBIC), Cordoba, Spain; 3 Department of Medicine, University of Cordoba, Cordoba, Spain; 4 CIBER Fisiopatologia Obesidad y Nutricion (CIBEROBN), Instituto de Salud Carlos III, Madrid, Spain; 5 Department of Biochemistry-Research, Hospital Universitario Ramon y Cajal, Instituto Ramon y Cajal de Investigacion Sanitaria (IRyCIS), Madrid, Spain; 6 TNO, Zeist, The Netherlands; 7 Nutrition and Genomics Laboratory, Jean Mayer United States Department of Agriculture Human Nutrition Research Center on Aging at Tufts University, Boston, MA USA; 8 Department of Clinical Investigation, Centro Nacional Investigaciones Cardiovasculares (CNIC), Madrid, Spain; 9 Department of Nutritional Genomics, Instituto Madrileno de Estudios Avanzados en Alimentacion, Madrid, Spain

## Abstract

This prospective study evaluated whether baseline cholesterol efflux is associated with future development of type 2 diabetes (T2DM) in cardiovascular patients. We measured cholesterol efflux in all CORDIOPREV study (NCT00924937) participants free of T2DM at baseline (n = 462) and assessed its relationship with T2DM incidence during a 4.5 years of follow-up. Cholesterol efflux was quantified by incubation of cholesterol-loaded THP-1 cells with the participants’ apoB-depleted plasma. Disposition index was estimated as beta-cell function indicator. During follow-up 106 individuals progressed to T2DM. The cholesterol efflux/apoA-1 ratio was inversely associated with T2DM development independently of traditional risk factors (model-1, OR: 0.647, 95%CI: 0.495–0.846), and after additional adjustment for glycaemic parameters (model-2, OR: 0.670, 95%CI: 0.511–0.878). When cumulative incidence of diabetes was analysed by quartiles of cholesterol efflux/apoA-I, incidence of T2DM was reduced by 54% in subjects who were in the higher cholesterol efflux/apoA-I quartile compared to subjects in the lowest quartile (p = 0.018 and p = 0.042 for model-1 and 2). Moreover, participants who were in the higher cholesterol efflux/apoA-I presented significantly higher disposition index (β = 0.056, SE = 0.026; p = 0.035). In conclusion, HDL-cholesterol efflux normalised to apoA-I was inversely associated with T2DM development in cardiovascular patients. This association was independent of several T2DM risk factors, and may be related to a preserved beta-cell function.

## Introduction

Type 2 diabetes mellitus (T2DM) and cardiovascular disease are among the main causes of disability and death worldwide and its prevention is one of the main targets of the World Health Organization^1^. Both are complex disorders, and their simultaneous presence considerably increases the risk of macrovascular complications and death as demonstrated by the high recurrence rate of major atherosclerotic complications (~6%/year) in type 2 diabetic patients with a prior cardiovascular event. Moreover, in diabetic patients with a previous heart attack, the 7-year incidence of subsequent myocardial infarction is more than double that of non-diabetic individuals with previous myocardial infarction^2^. Therefore, prevention of T2DM should be a priority in cardiovascular patients.

Epidemiological evidence supports an association between T2DM and insulin resistance with low high-density lipoprotein-cholesterol (HDL-C) and high triglycerides levels^3,4^. In subjects with T2DM, the reduction in HDL-C levels is explained by both down-regulation of hepatic apolipoprotein A-I (apoA‑I) transcription, and greater clearance of HDL-C from the circulation. Moreover, the functionality of apoA-I could be impaired by glycation of the lipoprotein, which would worsen the effects of low HDL-C levels in T2DM subjects^5^.

The causal direction between HDL-C levels and diabetes is unclear. Evidence suggest that low HDL-C levels may precede and predict T2DM conversion in prediabetic or healthy subjects^6,7^ and progression of glycaemia in those with established T2DM^8^. However, the independent predictive value of HDL-C levels for future development of T2DM is difficult to establish. Moreover, a recent Mendelian randomization study showed that genetically reduced isolated HDL-C does not associate with increased risk of T2DM^9^. These ambiguities support the need to investigate whether HDL functionality, measured as cholesterol efflux, would contribute to a better T2DM prediction, as it was demonstrated for cardiovascular disease^10–13^. Along these lines, Saleheen *et al*.^12^ recently found in the EPIC Norfolk study that HDL efflux capacity was significantly lower in T2DM than in non-diabetic subjects, although another recent study in the CODAM cohort found no association between HDL cholesterol efflux and glucose tolerance status^14^. Nevertheless, to the best of our knowledge, there are no prospective studies showing the influence of HDL functionality on T2DM incidence.

Therefore, we hypothesised that the capacity of HDL to accept cholesterol from peripheral tissues or cells could serve as a predictor of T2DM. This prospective population-based cohort study aims to evaluate whether baseline HDL cholesterol efflux (raw and normalised to apoA-I) is associated with future development of T2DM in patients with cardiovascular disease.

## Results

### Study participants

From the 436 non-diabetic subjects selected at baseline, 106 developed T2DM after a median follow up period of 4.5 years. Table 1 shows the baseline demographic and anthropometric characteristics of the study subjects at baseline. Participants who progressed to T2DM presented significantly higher BMI and waist circumference than the subjects who remained as non-diabetic.

**Table 1:** Baseline demographics and anthropometrics characteristics of the study subjects by glycaemic status at the end of the follow-up period.

	Remained as non-diabetic subjects	Progressed to diabetes subjects	*P*
n	330	106	
Age (years)	57.3 ± 9.5	58.8 ± 9.1	0.175
Men/Women (n)	282/48	88/18	0.536
Current smoking, n (%)	23 (7.2)	10 (9.7)	0.403
Current drinking, n (%)	203 (61.5)	69 (65.1)	0.565
Lipid medication use, n (%)	289 (87.6)	89 (84.0)	0.329
Hypertension, n (%)	211 (64.1)	72 (67.9)	0.558
Weight (kg)	82.6 ± 13.1	85.3 ± 15.2	0.080
BMI (kg/m^2^)	29.9 ± 4.1	31.2 ± 4.8	0.006
Waist circumference (cm)	101.7 ± 10.7	105.1 ± 11.3	0.005

Data are presented as mean ± SD for continuous variables or n (%) for categorical variables.

**Figure 1:**
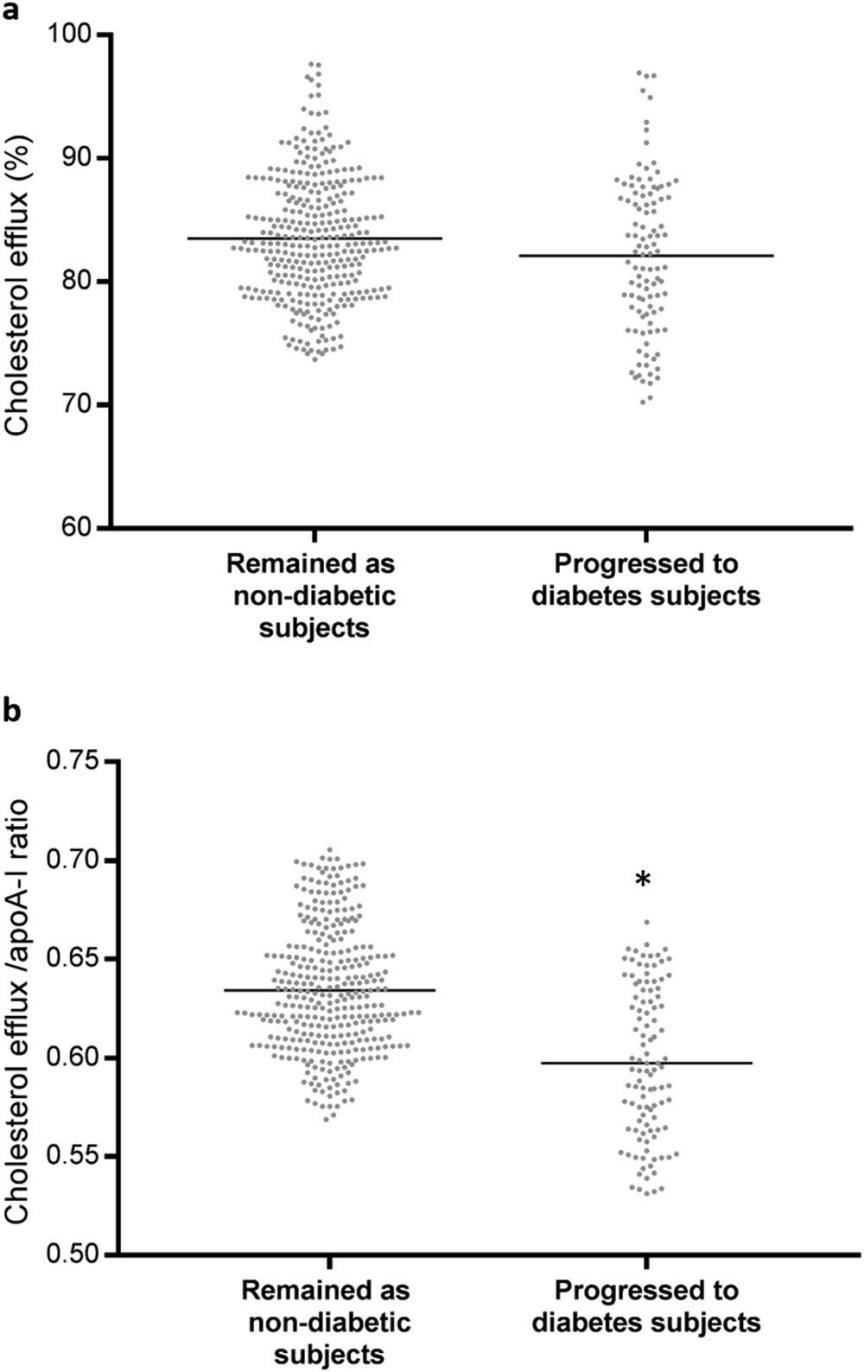
Cholesterol efflux and cholesterol efflux/apoA-I ratio by glycaemic status at the end of the follow-up period. Scatterplot showed raw data of cholesterol efflux in % (**a**) and cholesterol efflux normalised by apoA-I (**b**) in subjects who remained as non-diabetic and subjects that progressed to diabetes. Black bars indicated mean values in each group. *p < 0.005 between groups analysed by univariate model adjusted by age, sex BMI and batch number.

With respect to baseline glycaemic parameters (Table 2), patients that progressed to diabetes showed higher values of Hb1Ac, fasting glucose, insulin and HOMA-IR and lower disposition index than subjects who remained as non-diabetic. Table 2 also shows the lipid parameters at baseline. The only significant difference between the two groups was the significantly higher HDL-C/apoA-I ratio observed in subjects that remained as non-diabetic compared with those who progressed to diabetes (Table 2 and Fig. 1). We did not find differences in mean cholesterol efflux. However, when cholesterol efflux was normalised to apoA-I^15^, subjects who developed T2DM presented lower cholesterol efflux/apoA-I ratio than subjects that remained non-diabetic during follow-up (Table 2 and Fig. 1).

**Table 2:** Baseline glucose metabolism and lipid parameters of the study subjects by glycaemic status at the end of the follow-up period.

	Remained as non-diabetic subjects	Progressed to diabetes subjects	*P* ^a^
Hb1Ac (%)	5.85 ± 0.33	6.02 ± 0.33	<0.001
Fasting glucose (mmol/l)	5.12 ± 0.54	5.33 ± 0.60	0.006
Fasting insulin (pmol/l)	58.6 ± 40.3	72.4 ± 47.8	0.021
ISI	4.24 ± 2.50	3.49 ± 2.37	0.074
HOMA-IR	2.06 ± 1.74	3.36 ± 1.73	0.012
Disposition Index	0.99 ± 0.54	0.81 ± 0.47	0.006
Total cholesterol (mmol/l))	4.14 ± 0.77	4.28.3 ± 0.91	0.145
HDL-cholesterol (mmol/l))	1.15 ± 0.26	1.13 ± 0.27	0.640
LDL-cholesterol (mmol/l))	2.35 ± 0.65	2.42 ± 0.69	0.437
Serum triglycerides (mmol/l))	1.33 ± 0.65	1.49 ± 0.76	0.052
ApoA -I (g/l)	1.33 ± 0.21	1.36 ± 0.25	0.092
ApoB (g/l)	0.72 ± 0.13	0.75 ± 0.19	0.081
HDL-C/apoA-I ratio	0.33 ± 0.04	0.32 ± 0.04	0.025
Cholesterol efflux (%)^b^	83.5 ± 17.7	82.1 ± 19.0	0.192
Cholesterol efflux/apoA-I ratio	0.63 ± 0.12	0.60 ± 0.11	0.001

Data are presented as mean ± SD.

^a^ *P* between groups analysed by univariate model adjusted by age, sex BMI and batch number (in cholesterol efflux and cholesterol efflux/apoA-I ratio).

^b^Cholesterol efflux values are adjusted to the inter-assay control in each batch.

### Association of HDL-related parameters with T2DM development

**Figure 2:**
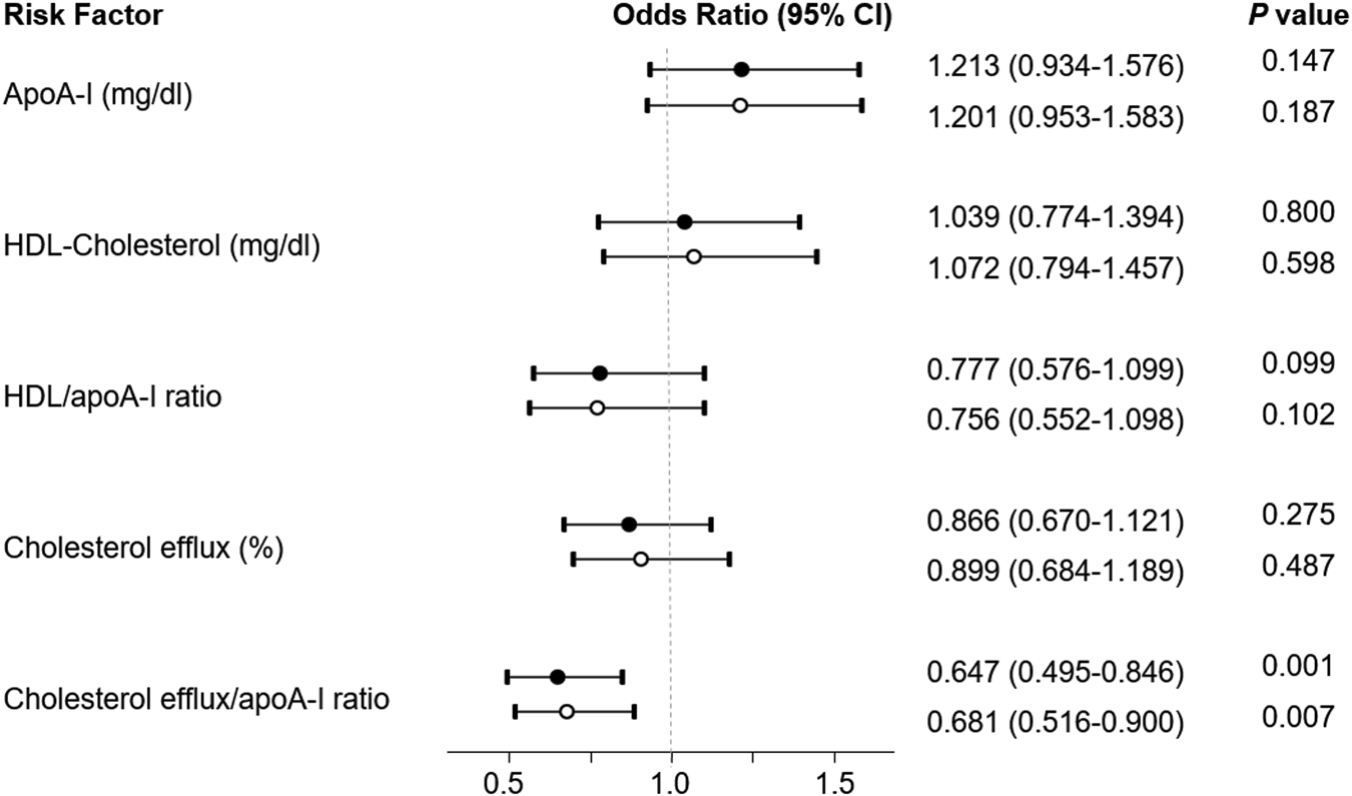
Odds ratio (95% CI) for T2DM. Odds ratio for continuous variables are per 1-SD increase. Logistic regression model 1 (●) adjusted by age, sex, BMI, smoking status, alcohol drinking, lipid-lowering treatment, hypertension, serum triglycerides and batch number (in case of cholesterol efflux). In model 2 (○) additional adjustment by HbA1c, HOMA-IR and Disposition Index was done.

We investigated the association of HDL-related parameters with T2DM (Fig. 2). Only cholesterol efflux/apoA-I ratio reached a significant association with T2DM development (*p* = 0.001). Neither cholesterol efflux, apoA-I, HDL-C nor HDL-C/apoA-I ratio were related to T2DM development. For a per-SD increase in cholesterol efflux/apoA-I ratio levels, the OR for T2DM was 0.647 (95% CI: 0.495–0.846, *p* = 0.001) in the first multivariate model adjusted for traditional metabolic risk factors (model 1). When we additionally adjusted the model for glycaemic parameters (model 2), cholesterol efflux/apoA-I ratio concentration remained independently associated with progression to T2DM (OR: 0.681, 95% CI: 0.516–0.900, *p* = 0.007). Since those subjects were in an intervention trial when they developed T2DM, a final adjustment of the model with the type of dietary intervention was done. Cholesterol efflux/apoA-I ratio levels still presented an independent association with T2DM development (OR: 0.669, 95% CI: 0.508–0.872, *p* = 0.005).

**Figure 3:**
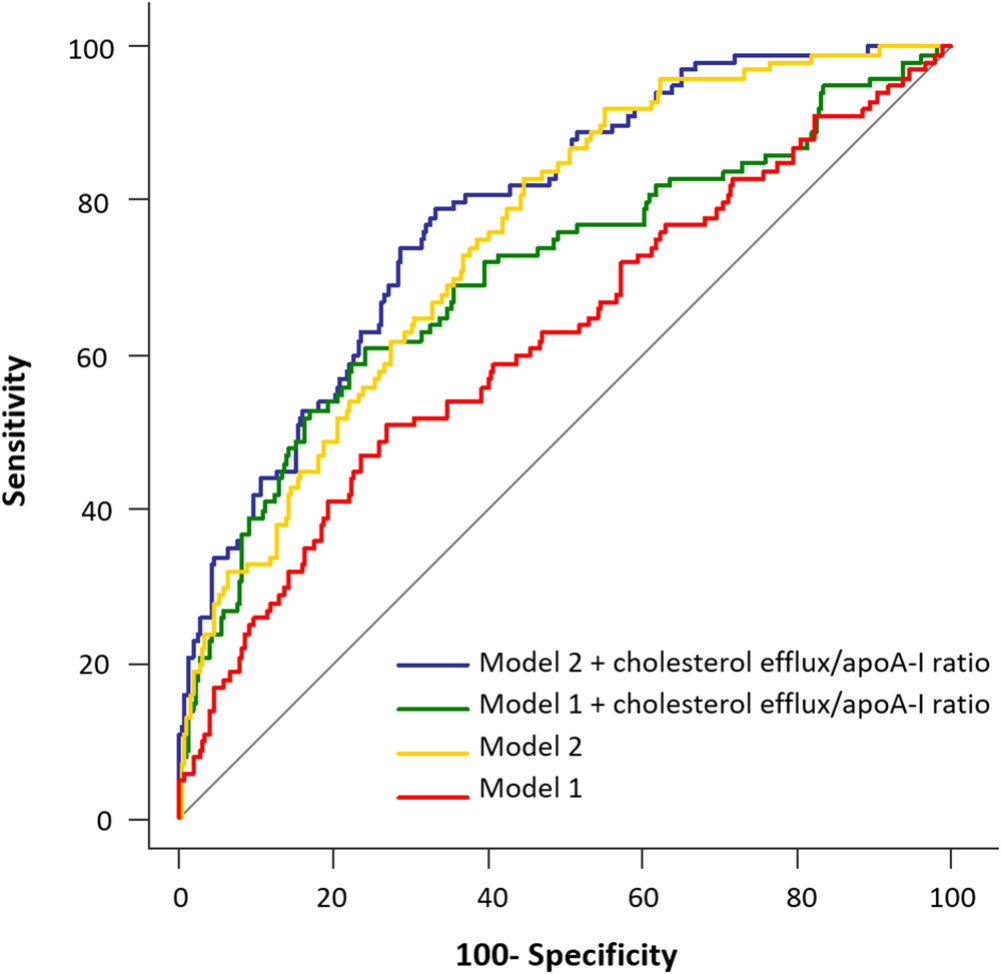
Receiver operator characteristic (ROC) curves from logistic regression models predicting T2DM. Model 1 (red line, AUC = 0.620) included the variables age, sex, BMI, smoking status, alcohol drinking, lipid-lowering treatment, hypertension, serum triglycerides and batch number. Model 2 (green line, AUC = 0.704) included the variables in model 1 plus glucose metabolism variables (Disposition Index, HOMA-IR and HbA1c). When cholesterol efflux/apoA-I ratio was added to both model 1 (yellow line, AUC = 0.753) and model 2 (blue line, AUC = 0.785), the prediction of both models increased significantly (p < 0.001).

The inclusion of cholesterol efflux/apoA-I ratio resulted in a very significant improvement of the overall performance of both logistic-regression models (Fig. 3). The AUC increased from 0.620 to 0.753 (*p* < 0.001) when cholesterol efflux/apoA-I ratio was added to the traditional metabolic risk factors multivariate model (model 1). The integrated discrimination improvement (IDI) measure for the cholesterol/efflux ratio was also significant (IDI = 0.115; *p* < 0.001) in the model 1 taking diabetes incidence as the main outcome. When cholesterol efflux/apoA-I ratio was included in the model which also included the glycaemic parameters (model 2), the increase in the AUC (from 0.714 to 0.782) was also significant (*p* < 0.001) and so the IDI (IDI = 0.090; *p* < 0.001).

**Figure 4:**
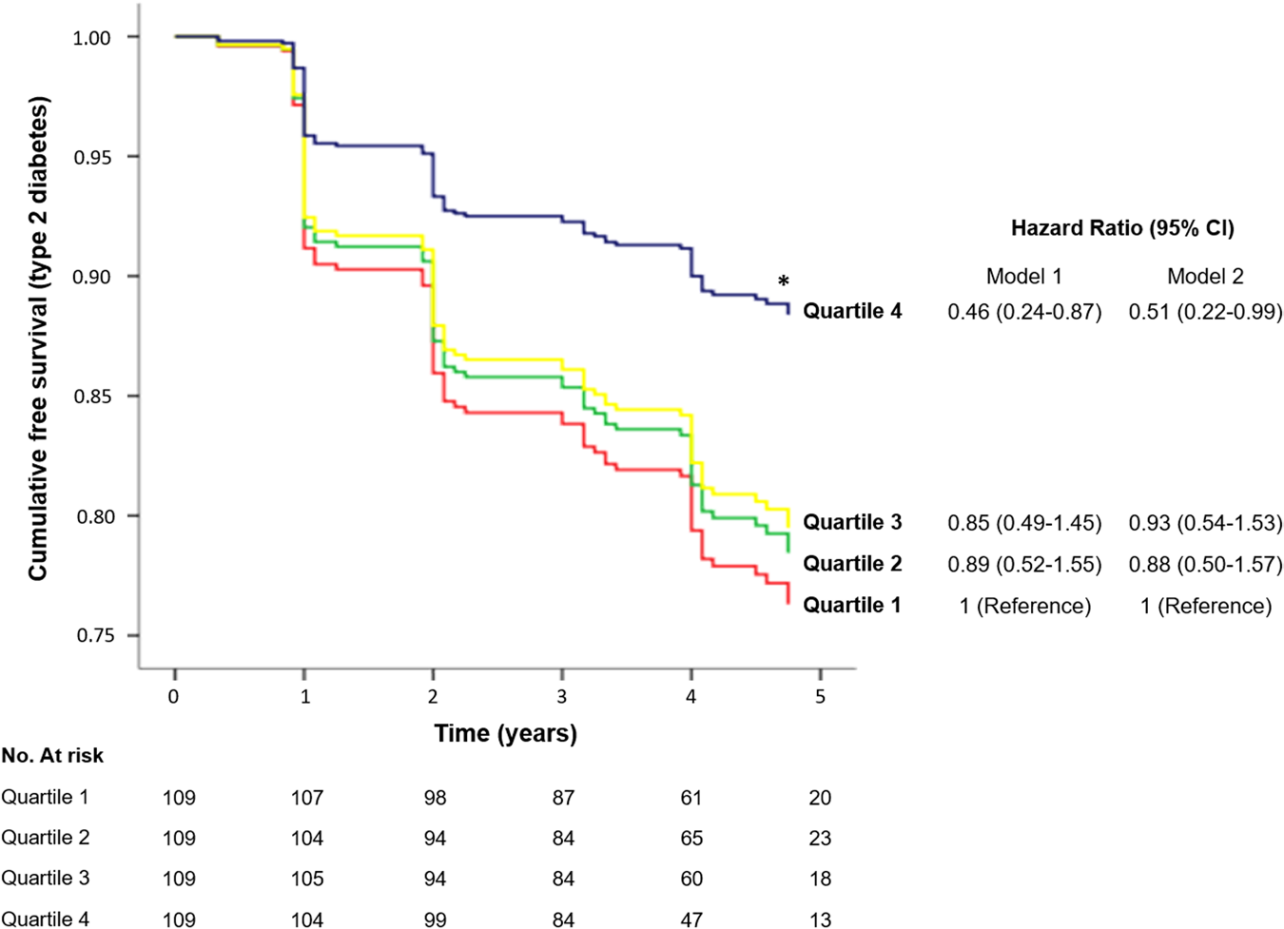
Cumulative T2DM free-survival by cholesterol efflux/apoA-I ratio quartiles. Cox regression models with outcome of T2DM are shown for quartiles of cholesterol efflux/apoA-I ratio, with the use of quartile 1 (subjects with the lowest cholesterol efflux capacity) as reference. Model 1 were adjusted by age, sex, BMI, smoking status, alcohol drinking, lipid-lowering treatment, hypertension, serum triglycerides and batch number. In Model 2, additional adjustment by Disposition Index, HOMA-IR and HbA1c were done. (*p < 0.05 quartile 4 vs quartile 1).

The rate of diabetes incidence per 1,000 person-years was 70.3 for the subjects who had the lowest cholesterol efflux/apoA-I ratio (quartile 1), 56.3 for the subjects in quartile 2, 67.1 for those in quartile 3, and 40.5 for the subjects who had the highest cholesterol efflux/apoA-I ratio (quartile 4). Cumulative incidence of diabetes was 27.2 (25.5–28.8), 22.3 (21.1–23.5), 24.6 (23.2–26.1) and 16.4 (15.7–17.1) for quartiles 1 to 4, respectively (Table 3). Figure 4 shows that cumulative diabetes-free survival was significantly lower in quartile 1 compared with quartile 4. After adjustment for several traditional risk factors (model 1), the development of diabetes was reduced by 54% in the subjects who had the highest cholesterol efflux/apoA-I ratio (quartile 4) compared to subjects in the first quartile (p = 0.018). Moreover, when we additionally adjusted the model for glycaemic parameters (model 2), the hazard ratio for T2DM of quartile 4 vs quartile 1 remained significant (*p* = 0.049).

**Table 3:** Cumulative incidence of T2DM by quartiles of cholesterol efflux/apoA-I ratio.

	Quartile 1	Quartile 2	Quartile 3	Quartile 4
	0.49 (0.17–0.55)^**a**^	0.58 (0.56–0.62)	0.66 (0.63–0.69)	0.76 (0.70–1.28)
Total sample size (n)	109	109	109	109
Person-years (n)	455.2	462.0	446.9	444.5
New cases of type 2 diabetes (n)	32	26	30	18
Rate per 1000 person-years	70.3	56.3	67.1	40.5
Cumulative incidence (95% CI)	27.2 (25.5–28.8)	22.3 (21.1–23.5)	24.6 (23.2–26.1)	16.4 (15.7–17.1)
Model 1 adjusted Hazard Ratio (95% CI)^b^	1 (Reference)	0.89 (0.52–1.55)	0.85 (0.49–1.45)	0.46 (0.24–0.87)*
Model 2 Adjusted Hazard Ratio (95% CI)^c^	1 (Reference)	0.88 (0.50–1.57)	0.93 (0.54–1.53)	0.51 (0.22–0.99)^†^

^a^Median (Intertertil range).

^b^Cox proportional hazards Model 1 adjusted by age, sex, BMI, smoking status, alcohol drinking, lipid-lowering treatment, hypertension, serum triglycerides and batch number.

^c^In model 2, additional adjustment by HbA1c, HOMA-IR and Disposition Index was done.

^*^ *p* = 0.018 quartile 4 vs quartile 1.

^†^ *p* = 0.049 quartile 4 vs quartile 1.

### Relationship between cholesterol efflux/apoA-I ratio and glycaemic parameters

In order to study possible relationships between cholesterol efflux/apoA-I ratio and glycaemic parameters at baseline, we performed linear regression in all the subjects (Table 4). We found that subjects who had higher cholesterol efflux/apoA-I ratio also presented significantly higher disposition index (β = 0.056, SE = 0.026; *p* = 0.035). This association persisted after adjustment by age, sex, BMI, smoking status, alcohol drinking, lipid-lowering treatment, and batch number (β = 0.061, SE = 0.027; *p* = 0.023). No associations between cholesterol efflux/apoA-I ratio and the other glycaemic parameters were found.

**Table 4:** Linear regression analysis of cholesterol efflux/apoA-I ratio with glucose metabolism parameters.

	Unadjusted model		Adjusted model^a^	
	β (SE)	*P*	β (SE)	*P*
Hb1Ac	−0.099 ± 0.140	0.479	−0.020 ± 0.017	0.250
Fasting glucose	0.299 ± 0.490	0.541	−0.160 ± 0.495	0.745
Fasting insulin	−0.480 ± 0.296	0.106	−0.577 ± 0.298	0.093
HOMA-IR	−0.110 ± 0.073	0.135	−0.193 ± 0.107	0.082
ISI	0.052 ± 0.125	0.677	0.128 ± 0.121	0.291
Disposition Index	0.056 ± 0.026	0.035	0.061 ± 0.027	0.023

Data are presented as β ± SE: Unstandardised coefficients ± Standard Error.

^a^Adjusted by age, sex, BMI, smoking status, alcohol drinking, lipid-lowering treatment, and batch number.

## Discussion

In this prospective study, we report that the HDL cholesterol efflux normalised to apoA-I was inversely associated with future development of T2DM in a cohort of cardiovascular patients free from T2DM at baseline. During follow up, diabetes risk was reduced by 54% in subjects with the highest cholesterol efflux per apoA-I ratio at baseline compared to those who had the lowest cholesterol efflux/apoA-I. This association was independent of several established risk factors and persisted even after adjusting for glucose metabolism parameters. Furthermore, we present results that support the hypothesis that HDL cholesterol efflux may be involved in maintaining beta cell function, since we found that subjects who had higher cholesterol efflux/apoA-I ratio also presented higher beta cell function.

The most established functional property associated with HDL is its capacity to stimulate efflux of free cholesterol from cells which, besides its atheroprotective action, may be involved in glucose metabolism^5^. In this regard, it has been shown that HDL from patients with insulin resistance and T2DM have lower cholesterol efflux capacity from macrophages^12,16^. In those studies, the decrease in HDL functionality was related to the pattern of diabetic dyslipidaemia, and was proposed to increase cardiovascular risk in these subjects^17^. However, some authors have suggested that a reduction in cholesterol efflux might exacerbate tissue insulin resistance and beta cell dysfunction^5^. Nevertheless, this mechanism and its chronology need to be demonstrated. Our study supports that HDL functionality is impaired in patients at risk of T2DM and precedes the manifestation of the disease. Moreover, we present new evidence supporting that cholesterol efflux is an independent risk factor for the development of T2DM. Therefore, we consider that our findings may have important clinical and public health application, since the development and clinical implementation of practical techniques to measure HDL functionality could improve T2DM prediction and prevention.

Interestingly, whereas cholesterol efflux per apoA-I ratio at baseline was different among subjects that progressed to diabetes and subjects that remained as non-diabetic during follow-up, that was not the case for HDL-C and apoA-I levels. These results suggest that the lower cholesterol efflux to apoB-depleted plasma observed in those subjects who developed T2DM may due to a decreased ability of HDL particles to promote cholesterol efflux, rather than to their number; although further analysis including the HDL particle concentration and size should be necessary to validate these findings. However, it has been proposed that alterations in glucose metabolism may affect HDL functionality in several ways. It was established that insulin resistance increases the production of small, dense, triglycerides-enriched HDL, which could be the result of enhanced cholesteryl ester transfer protein activity and subsequent TG hydrolysis by hepatic lipase^17^. It appears that this replacement of cholesteryl esters by TG in the HDL lipid core can alter the conformation of apoA-I and reduce its binding affinity, and therefore, reduce cholesterol efflux^18^. Although we have not conducted the proper analysis to confirm lipid changes in HDL, we found that subjects who developed T2DM presented lower HDL-C/apoA-I ratio at baseline than those who remained non-diabetic, which is a measure related to the size and density of the particle^19^. Another factor that could impair HDL functionality is the advanced glycation end products (AGEs)-modification of apoA-I, resulting from maintained hyperglycaemia^20^. In our study, subjects who developed T2DM presented higher HbA1c levels at baseline compared to those who remained non-diabetic, which is consistent with the well-known role of HbA1c as a diagnostic and predictive factor for T2DM^21^. In addition, since HbA1c is considered the prototype of early glycated protein^22^, this may reflect an increased AGE activity in the subjects who progressed to T2DM, and therefore may explain the lower capacity of HDL to promote cholesterol efflux from macrophages found in these subjects. However, we did not measure apoA-I glycation, so further studies should be done in order to confirm this hypothesis.

Alternatively, or in combination with the mechanisms suggested above, there is evidence of a role of HDL in glucose metabolism^5^. Lipid accumulation and lipotoxicity in pancreatic beta cells have been shown to inhibit insulin production and secretion^23^; so HDL cholesterol efflux may be involved in maintaining beta cell function^24^. In support of this hypothesis, we found that subjects with higher cholesterol efflux/apoA-I ratio also presented higher disposition index, a surrogate of beta cell function^25^. This is also consistent with the findings from Dullaart *et al*. showing a positive relation between HDL functionality and beta cell function in T2DM subjects, although in subjects with normal fasting glucose or impaired fasting glucose the differences were not statistically significant^26^. The fact that we were able to reveal a significant association between HDL cholesterol efflux/apoA-I ratio and beta cell function in subjects without established diabetes may be due to the higher statistical power of our study. However, we cannot discard the influence of the different methodologies they used to evaluate beta cell function^26^. Moreover, some pharmacological studies found that increasing plasma HDL and apoA-I levels improve glycaemic control in diabetic patients^27^; but, to the best of our knowledge, no similar findings to those have been reported in pre-diabetics or non-diabetics subjects. Therefore, our findings support future clinical trials that target HDL functionality in order to prevent T2DM.

Our analysis was based on a large sample size, taking into account the technical difficulty of the method to measure cholesterol efflux (by incubation of cholesterol-loaded THP-1 cells with the participants’ apoB-depleted plasma.) and the specificity of the studied population (non-diabetic cardiovascular patients). Another important strength is the high follow-up rate (94%) during the long time of follow-up (4.5 years) with high incidence of new T2DM cases, which provide high statistical power. There are, however, several limitations. First, the study sample consisted of white subjects with an average age close to 60 years with established cardiovascular disease and a higher rate of T2DM incidence, most of them under lipid-lowering medication. Therefore, we cannot generalise our results to clinically healthy individuals or to other age groups and ethnicities. Another point is that we analysed cholesterol efflux in a human model of macrophages (THP-1 cell line). Although the assay we conducted has been validated and it is considered the closest to the *in vivo* situation^28^, it assumes unidirectional flux and does not account for the relative contribution of the various processes involved in cholesterol efflux out of different cell types. Therefore, we can just hypothesise that our findings in THP-1 macrophages may apply to pancreatic beta cells. However, the current evidence suggest that the ATP-binding cassette transporter A1 pathway represents a key cholesterol transport system for beta cells^29^, as occurs in cholesterol-loaded human macrophages^30^. Also, it is important to point out that HDL has more functionalities which may also affect diabetes development (e.g. anti-inflammatory, antiproliferative, antioxidative)^5^. Finally, we consider that our findings should be interpreted within the context of the experimental limitations, so the causal nature of the relationship between cholesterol efflux and the development of T2DM remains uncertain and the potential mechanisms should be explored and validated in future studies.

In conclusion, our prospective study has shown that HDL cholesterol efflux normalised to apoA-I was inversely associated with future development of T2DM in a cohort of patients with cardiovascular disease free from T2DM at baseline. This association was independent of several established T2DM risk factors, included HbA1c levels, and may be related to a preserved beta cell function, measured by the disposition index. These findings reinforce the concept that assessment of HDL functionality may be more precise than HDL-C levels to predict not only coronary heart disease, but also other chronic diseases, like T2DM.

## Patients and Methods

### Study design and participants

We performed this study with all the non-diabetic subjects at baseline participating in the CORDIOPREV trial, a long-term secondary cardiovascular prevention dietary intervention trial involving 1002 patients. The eligibility criteria, design, and methods of the CORDIOPREV clinical trial have been reported elsewhere^31^, and the protocol was registered at Clinicaltrials.gov (NCT00924937) on June 18th, 2009. Briefly, the CORDIOPREV study included men and women between 20–75 years old who had a coronary event at least six months before of enrolment. Subjects were recruited between November 2009 and February 2012, mostly at the Reina Sofia University Hospital, Cordoba, Spain. At baseline and every year, patients passed both medical and dietary interviews and underwent a blood analysis and an oral glucose tolerance test (OGTT). The Clinical Trials Ethics Committee of the Reina Sofia University Hospital approved the CORDIOPREV trial protocol and all its amendments, which all follow the Helsinki declaration and good clinical practices. The experimental protocol conforms to international ethical standards and written informed consent was obtained from all the subjects.

From the 1002 subjects included in CORDIOPREV, we excluded 540 patients who had a medical record for diabetes, were receiving glucose-lowering treatment, and/or had some of the American Diabetes Association (ADA) criteria for the diagnostic of diabetes at baseline (fasting glucose ≥7 mmol/l, or 2 h glucose ≥11.1 mmol/l, or HbA1c ≥6.5%)^32^. Of the 462 subjects free for diabetes at baseline, we selected for cholesterol efflux assessment those participants who had at least one year of follow-up. Subsequently, 436 individuals were eligible for this analysis. All participants were of European ancestry.

### Biochemical determinations and anthropometric measurements

Blood samples were drawn after an overnight fast in EDTA tubes. Lipid parameters and glucose were measured in Architect c-16000 analysers (Abbott, Chicago, IL, USA) by spectrophotometric techniques (enzymatic colorimetric methods): hexokinase method for glucose, and oxidation-peroxidation for total cholesterol, HDL-C and serum triglycerides. LDL-cholesterol (LDL-C) was calculated using the Friedewald formula (provided the serum triglycerides level was below 300 mg/dl). ApoA-I and apoB were determined by immunoturbidimetry. HDL-cholesterol/apoA-I (HDL-C/apoA-I) ratio was calculated as a marker of HDL size. Plasma level of insulin was measured by chemiluminescent microparticle immunoassay (Abbott Architect, Chicago, IL, USA). HbA1c was determined by HPLC.

Patients also underwent a standard OGTT at baseline and every year. After an overnight fast, blood was sampled from a vein before oral glucose intake (0 min) and again after a 75 g flavoured glucose load (Trutol 75; Custom Laboratories, Baltimore, MD, USA). Blood samples were taken at 30, 60, 90 and 120 min to determine glucose and insulin concentrations^33^. Insulin resistance and beta cell function indices were estimate as previously reported^25^. Briefly: HOMA-IR was calculated as (fasting insulin (µU/ml) × fasting glucose (mmol/l)))/22.5; the insulin sensitivity index (ISI) = 10,000/√((fasting insulin (pmol/l) × fasting glucose (mmol/l)) × (mean OGTT insulin (pmol/l)) × (mean OGTT glucose (mmol/l))) the Disposition Index = ISI × (AUC30 min insulin/AUC30 min glucose), where AUC30 min is the area under the curve between baseline and 30 min of the OGTT for insulin (pmol/l) and glucose (mmol/l) measurements, respectively, calculated by the trapezoidal method.

T2DM diagnosis during follow-up was done by internal medicine physicians. They considered the subjects had developed T2DM if they started receiving glucose-lowering treatment, and/or had some of the American Diabetes Association (ADA) criteria for the diagnostic of diabetes during follow-up visits (every 12 months): fasting glucose ≥7 mmol/l, or 2 h glucose ≥11.1 mmol/l, or HbA1c ≥6.5%^32^.

Weight, height and waist circumference were measured according to standardised protocols, and the body mass index (BMI) was calculated as weight (kg)/height (m^2^). Systolic and diastolic blood pressure was measured with a validated digital automated blood pressure monitor. Hypertension was defined as systolic blood pressure ≥130 mmHg high blood and/or diastolic blood pressure ≥85 mmHg pressure and/or current use of antihypertensive drugs.

### Assessment of cholesterol efflux capacity

The macrophage-cholesterol efflux capacity was measured using a protocol based on methods previously described^34^ at baseline. Blood samples were collected in EDTA tubes after a 12 h fasting period. Plasma was separated by centrifugation at 4 °C and was immediately frozen at −80 °C until its use.

Efflux assays were performed using human THP-1 monocytes. For the assays, THP-1 cells were plated in 48 multi-well plates at a concentration of 125,000 cells/well and they were treated with phorbol 12-myristate 13-acetate (50 ng/mL) for 72 h to become fully differentiated macrophages. Then, THP-1 macrophages were labelled with 1.2 μCi/mL ^3^H-cholesterol and cholesterol loaded with 50 µg/mL of acetylated-LDL (acLDL) in RPMI medium containing 10% lipoprotein deficient FBS (density >1.21 g/L). After 24 h, cells were washed 3 times with PBS, 0.1% human serum albumin (HSA) to remove the excess of ^3^H-cholesterol and acLDL, and they were equilibrated in serum-free medium overnight at 37 °C. The next day, plasma samples were thawed and treated with polyethylene glycol to precipitate apoB-containing lipoproteins. Briefly, 40 parts polyethylene glycol solution (20% polyethylene glycol 8000 molecular weight in 200 mM glycine buffer, pH 7.4) was added to 100 parts plasma and mixed by pipetting, then incubated at room temperature for 20 min before spinning in a microcentrifuge at 10,000 rpm for 30 min at 4 °C. The supernatant, which contained the HDL fraction, was recovered. Finally, efflux medium containing 2% apoB-depleted plasma was added to THP-1 macrophages. The efflux period was 4 h, after which the medium was removed for quantifying the ^3^H^−^cholesterol present therein and in cells by scintillation counting. Each sample was run in triplicate, and within each plate were always included, besides the study subjects’ plasmas, serum-free medium containing 0.2% HSA and an inter-assay control (2%) consisting of pooled apoB-depleted plasmas from four healthy volunteers and stored at −80 °C.

We calculated percentage efflux to medium by the formula: (dpm ^3^H^−^cholesterol in the medium × 100)/(dpm ^3^H^−^cholesterol in the medium + dpm ^3^H^−^cholesterol in cells). The blank correction was done by subtracting the efflux of each sample to serum-free medium value in each batch. To standardise the percentage efflux obtained in the several analyses, we adjusted values for the study subjects to the inter-assay control in each batch as follows: (study subject cholesterol efflux × 100)/inter-assay control cholesterol efflux. Also, we normalised the HDL cholesterol efflux to apoA-I by calculating the cholesterol efflux/apoA-I ratio^14^: cholesterol efflux (%) divided by apoA-I (mg/dl). The inter-assay variability across plates was controlled by the inter-assay control. The inter-assay coefficient of variation was 6.4% and the intra-assay coefficient of variation was 4.5%.

### Statistical analysis

Normal distribution was tested for all measured variables, and serum triglycerides (TG), fasting insulin and the glucose metabolism indices were log-transformed. Statistical analysis was carried out using SPSS software version 21.0 for Windows (SPSS, Chicago, IL, USA). The data are presented as mean (SD) for continuous variables and as n (%) for categorical variables. We compared baseline characteristics between subjects that progressed to T2DM and those who remained as non-diabetic by univariate model analysis adjusted by age, sex, BMI and batch number (in case of cholesterol efflux variables).

Logistic regression models were fitted to test the different HDL-related variables in determination of the odds ratio (OR) of T2DM. Study-specific OR’s and 95% confidence intervals (CIs) were estimated for each continuous variable per 1-SD increase. Two separate multivariate adjustments were performed. Model 1 was adjusted by age, sex, BMI, smoking status, alcohol drinking, lipid-lowering treatment, hypertension, serum triglycerides and batch number (in the case of cholesterol efflux variables). In model 2, additional adjustment by HbA1c, HOMA-IR and Disposition Index was done. Model performance (C-statistic) was assessed according to discrimination by means of the area under the receiver-operating-characteristic curve (AUC). Evaluation of model predictive performance was done by two-paired comparison between the AUCs of the different models were performed by z test and IDI analysis, using the MedCalc Statistical Software version 13.3.3 (MedCalc Software, Ostend, Belgium; http://www.medcalc.org; 2016) and SPSS package, respectively.

Cox proportional-hazards models were used to assess the association between cholesterol efflux/apoA-I ratio and the time to a first event for T2DM. Multivariable adjustments were performed as indicated (model 1 and model 2). The exposure time was calculated as the time between the baseline visit and the date of T2DM diagnosis or the date of the last visit for fourth year of follow-up (October 7, 2014), whichever came first. The prespecified analyses calculated the hazard ratios (HRs) with 95% confidence intervals (CIs) of T2DM events associated with increasing quartiles of cholesterol efflux/apoA-I ratio. The proportional-hazards assumption was met for all models. Kaplan–Meier survival curves were plotted to estimate the probability of remaining free of T2DM during follow-up. Finally, associations between cholesterol efflux/apoA-I ratio and glycaemic parameters, as continuous dependent outcomes, were analysed by linear regression analysis. Then, further analysis adjusted by age, sex, BMI, smoking status, alcohol drinking, lipid-lowering treatment, and batch number were done.

### Data availability statement

Study protocol and statistical code: Available from Dr. Lopez-Miranda (email, jlopezmir@uco.es) Data set: Not publicly available.
